# Prevalence of asymptomatic malaria and associated factors among pregnant women at Boset District in East Shoa Zone, Oromia Region, Ethiopia: a cross-sectional study

**DOI:** 10.1186/s12936-023-04460-2

**Published:** 2023-01-25

**Authors:** Fufa Balcha, Takele Menna, Fantu Lombamo

**Affiliations:** 1 School of Public Health, Adama Hospital Medical College, Adama, Ethiopia; 2 School of Public Health, St Paul’s Hospital Millennium Medical College, Addis Ababa, Ethiopia

**Keywords:** Asymptomatic Malaria, Pregnancy, Associated factors, Prevalence, Ethiopia

## Abstract

**Background:**

Malaria infection during pregnancy is a significant public health problem that puts pregnant women at risk. Interruption of transmission of asymptomatic malaria among a population remained a challenge and the host serves as a reservoir for the malaria parasite; and is also recognized as a major barrier to malaria elimination. This study aimed to assess the prevalence of asymptomatic malaria and associated factors among pregnant women in the Boset District, East Shoa Zone, Oromia, Ethiopia.

**Methods:**

A community-based cross-sectional study was conducted to assess the prevalence of asymptomatic malaria and associated factors in pregnant women from February to March 2022. Using multistage sample techniques, 328 asymptomatic pregnant women were enrolled. Data were collected using a structured questionnaire. A rapid test and Giemsa-stained blood smear microscopy were used to diagnose *Plasmodium* infections. Epi info version 7 was used to code, enter, and clean data before being uploaded to SPSS version 25.0 for analysis. Bivariable and multivariable binary logistic regression were employed to find the associated factors. Variables in the multivariable model with a p-value < 0.05 were considered significantly associated with asymptomatic malaria.

**Results:**

Of the total 328 pregnant women who participated in this study, 9(2.74%) and 10(3.05%) were confirmed to be infected with *Plasmodium* species by microscopy and rapid diagnostic tests, respectively. Asymptomatic malaria during pregnancy was found to be significantly associated with not using an insecticide-treated bed net [(P = 0.002, AOR: 9.61; 95% CI (2.22–41.53)], lack of consultation and health education about malaria prevention during Antenatal care attendance [(P = 0.04, AOR: 4.05; 95% CI (1.02, 16.05)], and living close stagnant water [(P = 0.02, AOR: 4.43; 95% CI (1.17,16.82)].

**Conclusions:**

The current study showed that asymptomatic malaria is prevalent in pregnant women. Not using insecticide-treated bed nets, inadequate health education during antenatal care, and living close to stagnant water are significantly associated with malaria infection. Thus, using insecticide-treated bed nets, health education, and avoiding stagnant water from residential areas could play significant roles in preventing asymptomatic malaria among pregnant women in the study area.

## Background

Malaria is a disease caused by protozoan parasites of the genus *Plasmodium*, which are transmitted to humans by blood-feeding female Anopheles mosquitoes. *Plasmodium falciparum, Plasmodium vivax, Plasmodium ovale,* and *Plasmodium malariae* are the four human *Plasmodium* species [1]. *Plasmodium falciparum* is found in most of the tropical regions of the world and is the most dangerous of the five species in terms of both its lethality and morbidity [2].

Malaria infection during pregnancy is a significant public health problem that poses a significant risk to the pregnant woman, fetus, and newborn. In sub-Saharan Africa, malaria mainly affects children and pregnant women [3]. Although malaria is often asymptomatic in some regions of Africa during pregnancy, it nevertheless results in severe maternal anemia and low birth weight babies. Because low birth weight is so strongly correlated with a child's likelihood of surviving, successfully treating malaria during pregnancy could prevent 75,000–200,000 newborn fatalities annually [4]. Malaria during pregnancy affects the mother, fetus, and neonates [5]. It increases the risk of stillbirths, spontaneous abortion, premature delivery, and low birth weight [5, 6]. In areas with high transmission, malaria is often asymptomatic in adults. This is due to acquired immunity from constant exposure and previous childhood infections [7]. This immunity does not cover infection, but it does reduce the risk of serious illness [8]. The acquired immunity also protects pregnant women and malaria infections are often asymptomatic. However, pregnant women are still predisposed to malaria and the risk of severe symptomatic disease is higher than that of non-pregnant women [7, 8]. Infection with malaria during pregnancy has significant effects not only on the pregnant mother’s health, but also on her child’s birth outcomes. Nevertheless the acute illness due to malaria is uncommon; and the disease in many instances remains silent during pregnancy [9].

Malaria is one of the most widespread human parasitic diseases that ranks first in terms of its socio-economic and public health importance in the tropical and subtropical regions of the world, particularly in sub-Saharan African and Southeast Asian countries [2, 10]. As stated by World Health Organization (WHO), there were approximately 228 million cases of malaria worldwide, and the death toll reached 435,000 in 2018 [11].

In Ethiopia, an estimated 55.7 million people (68% of the population) are at risk of malaria, and three fourth of the landmass is considered malarious [12]. The Federal Ministry of Health (FMOH) estimates the annual cases of clinical malaria as 5–10 million, which corresponds to 12% of outpatient consultations and 10% of hospital admissions [13]. In the country, *P. falciparum* and *P. vivax* are the main species that account for about 60 and 40% of malaria cases, respectively [13], Recent reports indicate a shift in dominance from falciparum to vivax in highland areas [14].

Asymptomatic malaria is a new challenge to the national strategic plan for malaria prevention and control (2011–2015): a situation in which a human *Plasmodium* reservoir is maintained with individuals who are not treated because they are undiagnosed since they are asymptomatic [13]. The use of long-lasting insecticidal nets (LLINs), the administration of intermittent preventive treatment with artemisinin-based combination therapy (ACT), and adequate case management through rapid and effective therapy for malaria in pregnant women are the current strategies recommended by the WHO [15]. Currently, as part of the antenatal care (ANC) service package, WHO is working to improve access to ACT for pregnant women in all areas of moderate to high malaria transmission in Africa, but these activities are not used as ANC services to pregnant women in Ethiopia [15].

The presence of asymptomatic infections is less well-known in environments with pronounced seasonality [16]. However, in recent years such cases have also been reported from areas with low endemics such as the Amazon basin malaria, as the detection and treatment of all sources of infection is very important at this stage in Brazil, Peru [17], Colombia[18], Solomon Island [19], and Principe [20]. Any successful malaria elimination strategy depends on the ability to find and treat the asymptomatic reservoir. Globally, various studies reported the prevalence of asymptomatic malaria infection among pregnant women in both the control and eradication phases by taking into account the significance of asymptomatic malaria infection in diversified endemic regions [21].

In Ethiopia; a study on the prevalence of asymptomatic malaria in pregnant women; reported by different study groups shows a prevalence in the district of Merti, Oromia (3.6%) in 2016 [22]. Sanja (6.8%) in 2014 [23], and Arba Minch town (9.1%) in 2015 [24].

In both stable endemic regions and regions with the unstable transmission, asymptomatic malaria is frequently seen. Although acute malaria infections were given a lot of attention in many malaria-endemic regions of the world, asymptomatic malaria infection is yet to get due attention. In contrast, the asymptomatic host remained a significant barrier and challenge to the prevention, control, and eradication strategies of malaria in different countries of the world [25].

Therefore, this study aimed to assess the prevalence of asymptomatic malaria and associated factors among pregnant women in Boset District, Oromia Regional State, Ethiopia.

## Methods

### Study area

This study was conducted in Boset District which is located 125 kms southeast of Addis Ababa and has a total landmass of 14,050.27 square kilometres. It is one of the districts of the East Shewa Zone. The district is bordered on the south by the South Arsi Zone, the north by the North Amhara Regional State, the west by the West Adama District, and the east by the East Fentale District. The elevation of the District was 1050 m above sea level and 2500 m above mean sea level. The average annual rainfall and temperature range from 600 to 900 mm and 12 to 34 degrees Celsius, respectively. The district has three climate zones: 79 percent low-land "Kola," 20 percent mid-land "Woyinadega," and 1 percent high-land "Dega."

### Study design and period

A community-based cross-sectional study was conducted from February to March 2022.

### Population

#### Source population

The source population was all pregnant women living in the Boset district, East Shoa zone.

#### Study population

All pregnant women who live in selected Villages of Boset District.

### Inclusion and exclusion criteria

*Inclusion criteria:* All pregnant women with the absence of disease symptoms/signs of malaria within the past 48 h, axillaries temperature ≤ 37.5 ℃, and permanent residents in the study area.

*Exclusion criteria:* Pregnant women had taken antimalarial drugs in the last 4 weeks before the study period.

### Sample size determination and sampling technique

The sample size was calculated using the single population proportion sample size calculation formula, with a 95% confidence level, a 5% margin of error, and the proportion of asymptomatic malaria infection among pregnant women (P) taken as 11.2% [26]. The final calculated sample size was 328.

$$n=\frac{{{Z}_{\alpha /2}}^{2}P(1-P)}{{d}^{2}} =\Rightarrow n=\frac{{1.96}^{2}*0.112(1-0.112)}{{0.05}^{2}} = 152$$

$$152 + 8\% \left( {non - response~rate~} \right)x2~\left( {Design~Effect} \right) = 328$$

Key: n = sample size, P = proportion of asymptomatic malaria infection, and d = margin of error.

**Fig. 1:**
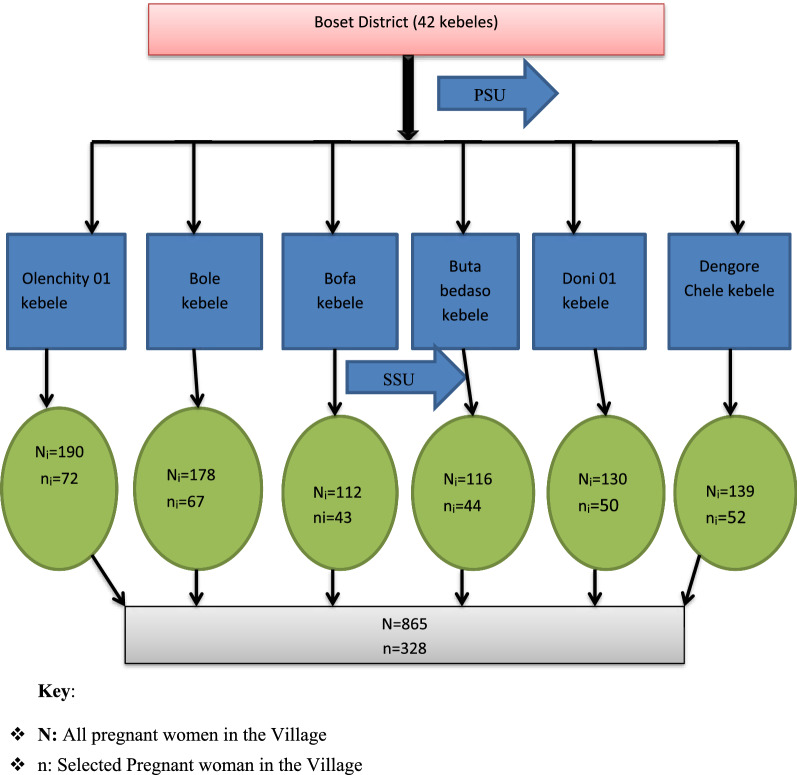
Flow chart indicating the sampling procedure for a study conducted on asymptomatic malaria among pregnant women in Boset District, Oromia, Ethiopia, 2022. Key: N: All pregnant women in the Village. n: Selected Pregnant woman in the Village

In addition, from the total 42 villages in the study area (Boset district) 6 villages were selected using the random sampling method. The above-calculated sample size was proportionally distributed to each village as per the current number of pregnant women population (Fig. 1). Then, a multistage sampling technique was employed to get each study subject during the actual data collection period.

### Study variables

*Dependent variable*: Asymptomatic malaria among pregnant women.

*Independent variable*: Age, residence, gestational age, previous infection with *Plasmodium*, living close to stagnant water, indoor residual spray, use of insecticide-treated bed net, gravidity, ANC attendance, trimester.

### Socio-demographic characteristics

Pre-tested and semi-structured questionnaires were administered by six trained Nurses and midwives interviewers to obtain data on socio-demographic characteristics and factors associated with asymptomatic malaria. They were supervised by one health professional with a qualification of BSc degree. The questionnaire was initially prepared in English and it was translated into Afan Oromo, the local language, and back into English by language experts, to check the consistency.

### Blood sample collection and processing

Capillary blood samples were collected by finger pricking using a disposable lancet. Giemsa-stained blood smear microscopy and RDTs were employed for the diagnosis of asymptomatic malaria parasitaemia at Olenchity Primary Hospital, Olenchity Health Center, and Bole Health Center. Asexual *Plasmodium* parasite density per microlitre (μl) of blood was determined by counting the number of parasites per 200 white blood cells on a thick blood film assuming a total standard WBC count of 8000/μl. The degree of parasite density was graded as mild, moderate, and severe when the counts will be between 1–999 parasites/μl, 1000–9999/μl, and > 10,000/μl, respectively, following the method described elsewhere [27].

$${\raise0.7ex\hbox{${{\text{Parasite}}}$} \!\mathord{\left/ {\vphantom {{{\text{Parasite}}} {{\text{ul}}}}}\right.\kern-\nulldelimiterspace} \!\lower0.7ex\hbox{${{\text{ul}}}$}} = \frac{{{\text{No}}{\text{. asexual stages x 8000 Leukocytes}}}}{{{\text{200 Leukocytes}}}}$$

### Operational definitions

*Asymptomatic malaria*: The absence of malaria-related symptoms within the past 2 days and at the time of the survey, and the presence of malaria parasites in the blood.

*Residents*: An individual that lives in a selected study village at least for 6 months before the start of the data collection time frame.

### Data quality assurance

#### Quality control

Two experienced laboratory technologists individually examined the microscopic slides. Hundred microscopic fields of the thick smear were examined before concluding as negative. The discrepancy between the first and second readings was settled by a third senior laboratory technologist. The manufacturer’s instructions were strictly followed for the RDTs. Blood smear microscopy readers were blinded to the result of RDTs.

#### Data quality management

All laboratory materials such as rapid test kits, slides, thermometers, and sample transporting systems were checked by experienced laboratory professionals. The specimens were also checked for serial number, quality, and procedures of collection. The laboratory professionals involved in RDT and light microscopy examination were trained in malaria diagnosis and quality assurance training. The rapid test kit was checked for the expiration date, correct collection procedures, and samples as well as inbuilt control appearances. Inconsistent results of light microscopy were checked again to confirm the findings.

### Data processing and analysis

Data were coded, entered into, and cleaned using Epi info version 7.2 and transferred to SPSS version 25.0 for analysis. Both descriptive and inferential statistics were employed for the analysis of data. Frequencies were used to determine the prevalence of asymptomatic *Plasmodium* infection in pregnant women. The Chi-square assumption was checked for all categorical independent variables. Both bi-variable and multi-variable logistic regressions were used to assess the association between outcome and explanatory variables. Factors with p-value ≤ 0.25 from the bi-variable model were included in the final model. Variables having a p-value < 0.05 from the multivariable model were considered as having a statistically significant association with the outcome. An adjusted Odds ratio with 95% CI was used as a measure of association. The model goodness of fit was assessed using the Hosmer–Lemeshow test.

## Results

### Socio-demographic, obstetric, and malaria prevention methods characteristics of the pregnant women

This study included a total of 328 pregnant women, with 252 (76.8%) of them living in rural areas. Participants in the study ranged in age from 16 to 36 years old, with a mean age of 25.52 ± 4.74 years. The majority of study participants were farmers 192(58.5%) and 314(95.7%) were married. About 219 (66.8%) of respondents owned at least one bed net, and 179 (54.6%) of them sleep under bed nets the previous night. Only 134 (40.9%), of the households, had indoor residual spraying (IRS) in the last 12 months. About 27.7% of the pregnant women were multigravidae, 47.3% of them were in their second trimester of pregnancy, and the majority 84.8% of them were following antenatal care (Table 1).

**Table 1:** Socio-demographic characteristics and malaria prevention methods in boset district, Ethiopia, 2022

Variables	Frequency	Percent (%)	Variables	Frequency	Percent (%)
Age groups in years			Residence		
15–19	38	11.6	Rural	252	76.8
20–24	102	31.1	Urban	76	23.2
25–29	121	36.9	Gravidity		
30–34	52	15.9	Multigravidae	91	27.7
≥ 35	15	4.6	Primigravidae	131	39.9
Occupational status			Secondigravidae	106	32.3
Daily wage laborer	51	15.5	Gestational age		
Farmer	192	58.5	1st trimester	104	31.7
Government	23	7.0	2nd trimester	155	47.3
Private	48	14.6	3rd trimester	69	21.0
Other	14	4.3	ANC Attendance		
Educational status			Yes	278	84.8
No formal education	102	31.1	No	50	15.2
Primary School	125	38.1	ITN Ownership		
Secondary School	74	22.6	Yes	219	66.8
College/University	27	8.2	No	109	33.2
Marital Status			ITN Utilization		
Single	2	0.6	Yes	179	54.6
Married	314	95.7	No	149	45.4
Separated	5	1.5	IRS Use(past 1yrs)		
Divorced	7	2.1	Yes	134	40.9
			No	194	59.1

### Prevalence of malaria infection among asymptomatic pregnant women

The prevalence of asymptomatic *Plasmodium* infection was 9(2.74%) [95% CI (1, 4.5)] and 10(3.05%) [95% CI (1.2, 4.9)] by using microscopy and RDTs, respectively. The density of parasitaemia, as determined from the thick blood smear, ranged from 790 to 5600 parasites /μl. The geometric mean of parasite density among the 7 parasitaemic pregnant women was 3528.6/μl [95% CI (3310–3750)]. The majority of the study participants who were diagnosed with malaria cases had moderate parasitaemia 5(71.4%), while 2(28.6%) had mild parasitaemia (Table 1).

### Factors associated with asymptomatic *Plasmodium* infection

Socio-demographic characteristics, obstetric characteristics, and other health-related factors were analyzed using bivariable binary logistic regression for the possible association. Then, gravidity, ITN utilization, ITN ownership, consultation about malaria prevention methods during ANC attendance, and Living close to stagnant water were variables that showed a possible association with *Plasmodium* infection at a P-value of ≤ 0.25 and fit for multivariable logistic regression analysis.

In multivariable logistic regression analysis, ITN utilization, consultation about malaria prevention methods during ANC attendance, and living close to stagnant water had a statistically significant association with asymptomatic *Plasmodium* species infection at P-value < 0.05 after adjusting for possible confounders. This study also found that pregnant women who did not utilize ITN had 9.61 times [AOR 9.61; 95% CI (2.22, 41.53)] higher chance of having asymptomatic *Plasmodium* infection than those who did. Pregnant women who lived near stagnant water had 4.43 times [AOR 4.43; 95% CI (1.17, 16.82)] greater odds of having asymptomatic *Plasmodium* infection than those who did not live near stagnant water. In addition, pregnant women who did not receive malaria prevention education during their ANC follow-up had a 4.05-fold higher risk of malaria infection than their counterparts [AOR: 4.05; 95% CI (1.02, 16.05)] (Table 2).

**Table 2:** Bivariable and Multivariable analysis of associated factors for asymptomatic *Plasmodium* species infection among pregnant women at Boset district, 2022 (N = 328)

Variables		Malaria status		COR (95% CI)	AOR (95% CI)	P-value
		Negative (%)	Positive (%)			
Gravidity	Secondigravidae	101(95.3%)	5(4.7%)	1	1	
	Multigravidae	88(96.7%)	3(3.3%)	0.68[0.16,2.96]	0.31[0.22,5.80]	0.24
	Primigravidae	129(98.5%)	2(1.5%)	0.23[0.04,1.34]	0.22[0.04,2.18]	0.06
Counseling at ANC attendance	Yes	272(97.8%)	6(2.2%)	1	1	
	No	46(92%)	4(8%)	3.94[1.07,14.51]	4.05[1.02,16.05]	0.04**
ITN ownership	Yes	210(95.9%)	9(4.1%)	1	1	
	No	108(99.08%)	1(0.92%)	0.21[0.02,1.72]	0.16[0.02,1.43]	0.10
ITN utilization	Yes	176(98.3%)	3(2.7%)	1	1	
	No	142(95.3%)	7(4.7%)	2.89[0.73,11.38]	9.61[2.22,41.53]	0.002**
IRS use in the last year	Yes	131(97.8%)	3(2.2%)	1	1	
	No	187(96.4%)	7(3.6%)	1.63[0.41,6.43]	2.70[0.65,11.17]	0.16
Living close to stagnant water	No	49(92.5%)	4(7.5%)	1	1	
	Yes	269(97.8%)	6(2.2%)	3.66[0.99,13.44]	4.43[1.17,16.82]	0.02**

## Discussion

This study assessed the prevalence of asymptomatic malaria infection and associated factors among pregnant women. The prevalence rates of asymptomatic *Plasmodium* infections among pregnant women were 2.74% [95% CI = (1.26, 5.14)] and 3.05% [95% CI = (1.47, 5.54)] using Giemsa-stained blood smear microscopy and RDT, respectively. These findings are lower when compared to the results reported previously from many similar studies conducted in different countries. For instance, the reported prevalence from the Republic of Congo was 7% [28]; the rural district surrounding Arbaminch town, Ethiopia was 9.1% [24]; the global scenario reported 10.8% [29]; the study from South-West Nigeria reported 7.7% [30]; a study from Southern Laos reported 8.3% [31], and the study from India reported 5.4% [32]. The low prevalence of asymptomatic malaria infection among pregnant women in this study could be due to the improvements in malaria control interventions in Ethiopia or the likely difference in malaria epidemiology among various study populations and areas; and the differences in climatic conditions or seasons where the data were collected from different parts of the world. In addition, the current study was conducted during the season in which the malaria prevalence rate is usually lower in the study area. This could be the possible explanation for the observed lower prevalence rate of asymptomatic malaria among pregnant women in this study.

Nevertheless, the prevalence of asymptomatic malaria infection documented in this study is comparable to the findings of the studies conducted in Bangladesh, which was reported as 2.3% among pregnant women [33], and in Merti district, Oromia, Ethiopia (3.6%) [22]. According to the findings of the current study, 77.78% of the cases were caused by *P. falciparum*. This result was almost in line with the study conducted in Ethiopia that reported 65–75% of malaria infections are caused by *P. falciparum* [34]. However, our result was higher than the study done in the Merti district, Ethiopia which was 46.2% [22]. This high proportion of *P. falciparum* in this study is a clear implication that there is a need for aggressive prevention and control of the disease, especially among pregnant women. Moreover, *P. falciparum* causes the most severe form of the disease and devastating complications not only to the mother, but also to the fetus. However, the proportion of malaria cases caused by *P. falciparum* in the study was lower than that of the WHO malaria 2017 report, which stated that *P. falciparum* was responsible for almost 99% of malaria cases [2]. The cause for these variations might be owing to significant seasonal, inter-annual, and geographical variability. Large climate changes (temperature, rainfall, and relative humidity), human habitation, and population mobility patterns could be also the possible reasons for the observed variations in prevalence rates.

In addition, the findings revealed not using ITN effectively increased the odds of developing malaria infection during pregnancy. This finding is in agreement with the findings of previous studies conducted elsewhere. For instance, the studies from Malawi [35], Arbaminch, Ethiopia [24], and Sherkole district, West Ethiopia [36], reported that using bed nets significantly reduced malaria infection. These associations could be explained by the fact that ITNs successfully minimize human-mosquito contact, which in turn helps in preventing malaria illnesses.

Furthermore, the pregnant women who lived near stagnant water were more likely to have an asymptomatic malaria infection than their counterparts. This finding is also in agreement with the findings of research that were undertaken in the West Arsi Zone, Ethiopia [37], and the Southwestern part of Ethiopia [38]. This could be because stagnant water is the ideal site for malaria vector breeding, and those who live near stagnant water are more susceptible to the disease. According to the findings of this research getting a consultation and health education about malaria prevention methods during ANC follow-up significantly reduced the risk of asymptomatic malaria infection among pregnant women. In the studies conducted in other localities of Ethiopia, similar findings were discovered [36, 39]. In general, the effective use of various malaria prevention and control measures among different population segments also play vital roles in the prevention and control of asymptomatic malaria among pregnant women.

The main limitations of the study include the inability to identify a direct temporal association between asymptomatic malaria infection and its likely risk factors because of being a cross-sectional study design; in the inability to diagnose all subjects using real-time PCR due to resources constraints; and fact that the data were collected during the season with low transmission probability which could have underestimated the prevalence of asymptomatic malaria among the study population.

## Conclusions

This study showed that asymptomatic malaria is prevalent among pregnant women in the study area. *Plasmodium falciparum* is most prevalent species in the area. Inadequate use of ITNs, living close to stagnant water, and inadequate consultation and health education about malaria prevention methods during ANC attendance were found to be factors associated with the occurrences of asymptomatic malaria infection among pregnant women.

Thus, using insecticide-treated bed nets effectively, adequate consultation and health education on various malaria preventive measures, and avoiding stagnant water from residential areas could play significant roles in preventing asymptomatic malaria among pregnant women in Ethiopia in general and in the study area in particular.

## Data Availability

The data upon which the result is based could be accessed as a reasonable request.

## Abbreviations

ACT: Artemisinin-based combination therapy
AOR: Adjusted Odd Ratio
ANC: Antenatal Care
COR: Crude Odd Ratio
CI: Confidence Interval
EDTA: Ethylene Diamine Tetra Acetic acid
EFETP: Ethiopian Field Epidemiology Training Program
FMOH: Federal Ministry of Health
IRB: Institutional Review Board
IRS: Indoor Residual Spray
ITN: Insecticide-Treated Net
LLINs: Long-Lasting Insecticidal Nets
PCR: Polymerase Chain Reaction
PI: Principal Investigator
RDT: Rapid Diagnostic Test
SOP: Standard Operating Procedures
SPSS: Statistical Package for Social Science
SPHMMC: St. Paul’s Hospital Millennium Medical College
WBC: White Blood Cell
WHO: World Health Organization
